# Comparison of the phylogenetic analysis of PFGE profiles and the characteristic of virulence genes in clinical and reptile associated *Salmonella* strains

**DOI:** 10.1186/s12917-019-2019-1

**Published:** 2019-09-02

**Authors:** Bartłomiej Dudek, Marta Książczyk, Eva Krzyżewska, Klaudia Rogala, Maciej Kuczkowski, Anna Woźniak-Biel, Kamila Korzekwa, Agnieszka Korzeniowska-Kowal, Radosław Ratajszczak, Alina Wieliczko, Jacek Rybka, Gabriela Bugla-Płoskońska

**Affiliations:** 1 Department of Microbiology, Faculty of Biological Sciences, University of Wrocław, 51-148 Wrocław, Poland; 2 Department of Immunology of Infectious Diseases, Hirszfeld Institute of Immunology and Experimental Therapy, Polish Academy of Sciences, 53-114 Wrocław, Poland; 3 Department of Epizootiology and Clinic of Birds and Exotic Animals, Wrocław University of Environmental and Life Sciences, 50-366 Wrocław, Poland; 4 Wrocław ZOO, 51-618 Wrocław, Poland

**Keywords:** *Salmonella*, RAS, PFGE profiles, Zoonotic potential

## Abstract

**Background:**

*Salmonella* is generally considered as a human pathogen causing typhoid fever and gastrointestinal infections called salmonellosis, with *S.* Enteritidis and *S.* Typhimurium strains as the main causative agents. *Salmonella enterica* strains have a wide host array including humans, birds, pigs, horses, dogs, cats, reptiles, amphibians and insects. Up to 90% of reptiles are the carriers of one or more serovars of *Salmonella.* Extraintestinal bacterial infections associated with reptiles pose serious health threat to humans. The import of exotic species of reptiles as pet animals to Europe correlates with the emergence of *Salmonella* serotypes, which not found previously in European countries. The presented study is a new report about *Salmonella* serotypes associated with exotic reptiles in Poland. The goal of this research was to examine the zoonotic potential of *Salmonella* strains isolated from reptiles by comparative analysis with *S.* Enteritidis strains occurring in human population and causing salmonellosis.

**Results:**

The main findings of our work show that exotic reptiles are asymptomatic carriers of *Salmonella* serovars other than correlated with salmonellosis in humans (*S*. Enteritidis, *S*. Typhimurium). Among the isolated *Salmonella* strains we identified serovars that have not been reported earlier in Poland, for example belonging to subspecies *diarizonae* and *salamae*. Restriction analysis with Pulsed-field Gel Electrophoresis **(**PFGE), showed a great diversity among *Salmonella* strains isolated from reptiles. Almost all tested strains had distinct restriction patterns. While *S*. Enteritidis strains were quite homogeneous in term of phylogenetic relations. Most of the tested VGs were common for the two tested groups of *Salmonella* strains.

**Conclusions:**

The obtained results show that *Salmonella* strains isolated from reptiles share most of virulence genes with the *S.* Enteritidis strains and exhibit a greater phylogenetic diversity than the tested *S.* Enteritidis population.

**Electronic supplementary material:**

The online version of this article (10.1186/s12917-019-2019-1) contains supplementary material, which is available to authorized users.

## Background

*Salmonella* is a Gram-negative bacteria responsible for a wide variety of infectious diseases: typhoid fever, gastroenteritis, food poisoning and septicaemia. Multiple genes required for full virulence of *Salmonella* strains are encoded on *Salmonella* pathogenicity islands (SPI) and could be acquired by horizontal transfer from other organisms. Infections, caused by nontyphoidal *Salmonella* strains (NTS), are recorded worldwide (94 million cases/year) but the epidemiological data are probably underestimated as many milder cases are neither diagnosed nor reported [1]. Currently, there are more than 2600 *Salmonella enterica* serovars [2] identified up to date. *Salmonella enterica* subsp. *enterica* Enteritidis (*S*. Enteritidis) is the most commonly isolated serovar in Europe. Although the number of cases of salmonellosis has been decreasing for over a dozen of years, the epidemiological data from Poland indicate a significant increase of *Salmonella* gastrointestinal infections in 2017. The National Institute of Public Health reported 8652 cases of *Salmonella* infections in 2015 and 10,007 cases in 2017 [3]. A multitude of publications worldwide indicate the specific virulence of *S*. Enteritidis and its ability to induce acute systemic infections, that often lead to death of the patient [4–7]. Roll et al. reported a case of transplacental infection of a premature infant by NTS in a woman with diarrhea and fever. In the 29th week of pregnancy, after a caesarean section, the newborn died from a septic shock. The same *S.* Enteritidis strain was cultured from blood cultures of the premature infant and from samples collected from the mother (placenta, uterus) [5]. Pumberger and Novak reported a case of a lethal infection of a newborn caused by NTS. One day after the birth, the condition of the newborn has deteriorated rapidly, acute abdominal inflammation and bloody diarrhea occurred. During epidemiological investigation the same *S*. Enteritidis strain has been cultured from maternal vaginal and fecal swabs, which clearly indicated the vertical transmission of the *S*. Enteritidis during childbirth [4].

*Salmonella enterica* strains have a wide host range including humans, birds, pigs, horses, dogs, cats, reptiles, amphibians and insects [8]. Up to 90% of reptiles are the carriers of one or more serovars of *Salmonella*, rarely demonstrating symptoms of any disease [9–11]. The disease can develop as a result of stress, exposure to low ambient temperatures or a sudden change of diet.

A large number of human salmonellosis cases have been linked to zoonotic transmission from snakes, turtles, lizards and terrapins [12–15]. Reptile-associated salmonellosis (RAS) is a serious health problem, especially in countries where reptiles are kept as pets, and is usually recorded in children under 5 years of age and people with immunodeficiency [16]. Recently the term reptile-exotic-pet associated salmonellosis (REPAS) is used instead of RAS, as salmonellosis can be more and more often caused by the contact with exotic species of reptiles imported to Europe from other parts of the world rather than by the contact with native species [16, 17]. Most data about RAS come from the USA but this new epidemiological problem starts to affect more and more countries. The number of RAS cases in USA is about 74,000 per year. A study published in 2013, shows an upward trend in the number of RAS cases in children under 3 years of age in countries of the European Union in 2007–2010 [17]. Van Meervenne et al. reported a case of RAS in a 2-month-old baby which led to sepsis and meningitis [13]. Also Schneider et al. described a case of RAS in a 10-month-old baby which leads to septic arthritis [14]. There are also cases of RAS reported in adults [15]. *S. arizonae*, *S. diarizonae*, *S*. *houtenae*, *S.* Java, *S.* Poona, *S.* Pomona, *S*. Stanley, *S*. Minnesota and *S.* Chameleon are the most commonly reported *Salmonella* strains encountered in RAS [9, 18].

Subspecies *S. houtenae*, *S. arizonae* and *S. diarizonae* belong to the somatic group O48 which have been previously shown to contain sialic acid (NeuAc) in the O-specific polysaccharide [19–21]. NeuAc is an important component of the bacterial cell wall because of the participation in the phenomenon of molecular mimicry*. Salmonella* strains belonging to the O-antigen somatic group O48 are described in the literature as an important etiological factor causing acute gastroenteritis in children [22, 23]. The presence of sialylated structures on the bacterial cell surface is a part of defense mechanism against the host immune system [24–26]. In our previous studies we investigated the role of lipopolysaccharide and outer membrane proteins of *Salmonella* O48 strains in the generation of serum resistance [27–29].

The analysis of *Salmonella* strains isolated from exotic reptiles are not frequent in Poland [30, 31]. The aim of this study was the examination of the prevalence of *Salmonella* strains isolated from different species of reptiles and the determination of their potential of pathogenicity and the comparison with the strains occurring in human population in Poland. Isolated *Salmonella* strains were screened for the presence of sialic acid in the lipopolysaccharide molecule. The study aimed also to compare the potential virulence of these strains by determining the genetic diversity of virulence genes of already extensively distributed *S*. Enteritidis strains isolated from humans and *Salmonella* strains isolated from reptiles, which may be associated with an epidemiological problem.

Zoological gardens with exotic animals species are often involved in education in the field of species protection, including the protection of biological diversity. The zoological garden in Wrocław participates, among others, in the program of research and protection of the Komodo dragons in the Wae Wuul nature reserve situated on the west coast of the island of Flores in Indonesia. Reptile microbial research, as a part of biological biodiversity studies, indicates the importance of public knowledge in the context of epidemiological threats resulting from breeding reptiles at home. In this case the cooperation between zoological gardens and scientists turned out to be very fruitful.

## Results

### Identification of bacterial strains

Bacterial strains used in this study were assigned to *Salmonella* genus with the following methods: biochemical tests, MALDI-TOF MS analysis and 16S rRNA sequencing.

All tested bacterial strains were classified as *Salmonella enterica* group with 98.32–100% sequence similarity. MALDI-TOF MS identified the tested bacterial samples as *Salmonella* species. All thirty strains assigned as *Salmonella* were additionally serotyped and classified according to the Kauffmann-White-Le Minor scheme (Table 1).

**Table 1:** Tested *Salmonella* strains

Number of PCM^a^ strain	Origin	*Salmonella enterica* taxonomic classification
2817	human feces	Enteritidis
2935	human feces	Enteritidis
2814	human feces	Enteritidis
2936	human feces	Enteritidis
2812	human feces	Enteritidis
2937	human feces	Enteritidis
2808	human feces	Enteritidis
2810	human feces	Enteritidis
2811	human feces	Enteritidis
2938	human feces	Enteritidis
2939	human feces	Enteritidis
2940	human feces	Enteritidis
2815	human feces	Enteritidis
2941	human feces	Enteritidis
2816	human feces	Enteritidis
2942^b^	Flat-headed rock agama *(Agama mwanzae)*	*diarizonae* 61: c: z35
2943	Common collared lizard *(Crotaphytus collaris)*	Muenchen (O8)
2944^b^	Baja blue rock lizard *(Petrosaurus thalassinus)*	*diarizonae* 65: k: z
2945^b^	Leopard gecko *(Eublepharis macularius)*	*salamae* 30: 1, z28: z6
2946	Red-headed rock agama *(Agama agama)*	Hvittingfoss (O16)
2947^b^	Western bearded anole *(Anolis barbatus)*	16: m, t: -
2948	Radiated tortoise *(Astrochelys radiata)*	Amsterdam (O3)
2949^b^	Central bearded dragon *(Pogona vitticeps)*^c^	*salamae* 40: g, m, t: -
2950	Eastern kingsnake *(Lampropeltis getula)*	Adelaide (O35)
2951	Leopard tortoise *(Psammobates pardalis)*	*salamae* 18: z4, z23: -
2952	Garter snake *(Thamnophis sp.)*	*diarizonae* 53: z 10: z35
2953	Chinese water dragon *(Physignathus cocincinus)*	Virchow (O7)
2954	Central bearded dragon *(Pogona vitticeps)*^c^	Kaneshie (O42)
2955	Marginated tortoise *(Testudo marginata)*	Amsterdam (O3)
2956	Central bearded dragon *(Pogona vitticeps)*^c^	*diarizonae* 6: r: z

^a^ Polish Collection of Microorganisms (PCM)

^b^ *Salmonella* serovars first time isolated in Poland (based on available data) [31–34]

^c^ *Salmonella* strains isolated from the same animal

### Virulence genotyping

**Fig. 1:**
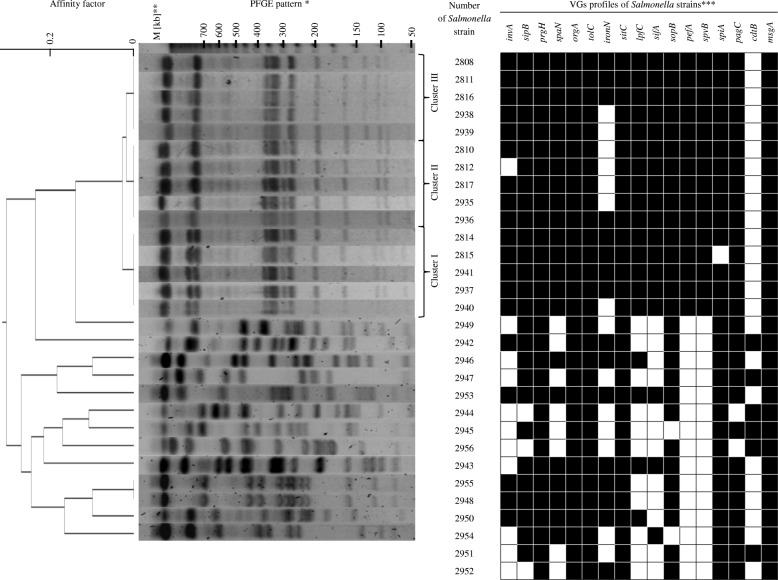
Genetic relatedness and VGs profiles of *Salmonella* strains isolated from humans and reptiles. * Strains PCM 2951 and PCM 2952 were nontypeable with PFGE method. ** Molecular Weight Marker. *** White square - lack of virulence gene, black square - presence of virulence gene

**Fig. 2:**
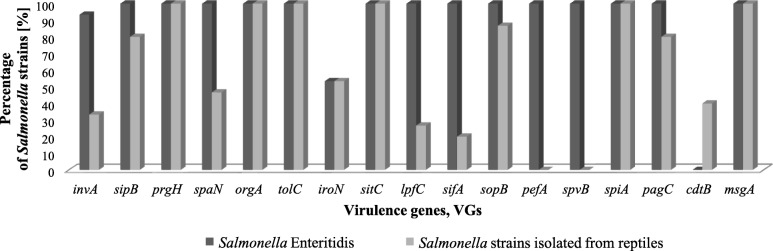
Percentage layout of detected VGs among *Salmonella* strains isolated from humans and reptiles

Results of virulence-associated genes (VGs) are presented in Fig. 1 and Additional file 1. Determined VGs profiles for both: *S.* Enteritidis from humans and *Salmonella* strains from reptiles, revealed differences in the prevalence of virulence genes. *Salmonella* strains from humans showed higher prevalence of VGs in comparison to reptilian *Salmonella* strains. The comparison of percentage prevalence of VGs among both investigated groups of *Salmonella* strains is presented in Fig. 2.

Following virulence genes were detected in genomes of all tested *S.* Enteritidis strains: *sipB, prgH, spaN, orgA, tolC, sitC, lpfC, sifA, sopB, pefA, spvB, spiA, pagC* and *msgA.* 93% of human *Salmonella* strains possessed *invA* gene and 53% of these strains had *iroN* gene. No one of the tested *S.* Enteritidis strains had the *cdtB* gene.

All *Salmonella* strains (100%) isolated from reptiles possessed in their genomes following virulence genes: *prgH, orgA, tolC, sitC, spiA* and *msgA* like as among *S.* Enteritidis strains. Also 53% of reptilian *Salmonella* strains had *iroN* gene like in the tested *S.* Enteritidis strains. The prevalence of *invA* (33%), *sipB* (80%), *spaN* (47%), *lpfC* (27%), *sifA* (20%), *sopB* (87%), *pagC* (80%) was lower than among human strains. Gene *cdtB*, which was not present in *S.* Enteritidis strains, was detected in 40% of reptilian *Salmonella* strains. However none of the tested *Salmonella* strains isolated from reptiles had *spvB* and *pefA* genes in its genome. Strains PCM 2942, PCM 2944, PCM 2956 and PCM 2952 belong to the same subspecies: *Salmonella diarizonae,* but all of these four strains had different VGs profiles. Strains PCM 2945, PCM 2949 and PCM 2951 were assigned to subspecies *Salmonella salamae* and their VGs profiles were similar with slight differences, strains PCM 2948 and PCM 2955 - both *S.* Amsterdam - had identical VGs profiles.

Analysis of the restriction profiles connected with Pulsed-field Gel Electrophoresis (PFGE) revealed that tested *S.* Enteritidis population is quite homogeneous in term of phylogenetic relations (results presented on Fig. 1). At the PFGE phylogenetic dendrogram human *Salmonella* strains are divided into three clusters. Among the strains in each cluster there are few slight differences in their VGs profiles (Fig. 1). In cluster I, the main VGs pattern was: *invA+, sipB+, prgH+, spaN+, orgA+, tolC+, iroN +, sitC+, lpfC+, sifA+, sopB+, pefA+, spvB+, spiA+, pagC+, cdtB-* and *msgA+,* however strain PCM 2940 was lacking one gene more *iroN.* In cluster II the leading VGs pattern is: *invA+, sipB+, prgH+, spaN+, orgA+, tolC+, iroN -, sitC+, lpfC+, sifA+, sopB+, pefA+, spvB+, spiA+, pagC+, cdtB-* and *msgA+,* but strain PCM 2812 was additionally lacking *invA* gene and strain PCM 2936 had in genome gene *iroN.* In cluster III, the strains PCM 2816, PCM 2808 and PCM 2811 had following VGs profiles: *invA+, sipB+, prgH+, spaN+, orgA+, tolC+, iroN +, sitC+, lpfC+, sifA+, sopB+, pefA+, spvB+, spiA+, pagC+, cdtB-* and *msgA+.* Strains PCM 2938 and PCM 2939 from cluster III was lacking *iroN* gene in comparison to strains PCM 2816, PCM 2808 and PCM 2811.

### Analysis of the phylogenetic relationship

Analysis of the restriction profiles of *Salmonella* strains isolated from reptiles revealed a great diversity among bacterial strains. Strains PCM 2951 and PCM 2952 were nontypeable with PFGE method, no restriction pattern of these strains were obtained despite of carrying out several experiments. Only two strains: PCM 2955 and PCM 2948 of the same serotype - *S.* Amsterdam, showing the same pattern of restriction, had also the same VGs profile: *invA+, sipB+, prgH+, spaN+, orgA+, tolC+, iroN +, sitC+, lpfC-, sifA-, sopB+, pefA-, spvB-, spiA+, pagC+, cdtB-* and *msgA+.* These fact indicate that strains PCM 2948 and PCM 2955 could be the same bacterial clone*.* Other *Salmonella* strains isolated from reptiles showed various patterns of restriction fragments and also had different VGs profiles (Fig. 1). Strains belonging to the subspecies *S. diarizonae*: PCM 2942 and PCM 2944, *S. salamae:* PCM 2945 and PCM 2949 had distant positions on the obtained phylogenetic tree. Three of reptilian *Salmonella* strains: PCM 2949, PCM 2954 and PCM 2956, isolated from the same animal (*Pogona vitticeps*), had completely different PFGE restriction profiles and differed also in VGs patterns. We did not observed any similarity between restriction profiles of *Salmonella* strains isolated from humans and reptiles.

### GLC MS/MS analysis

**Fig. 3:**
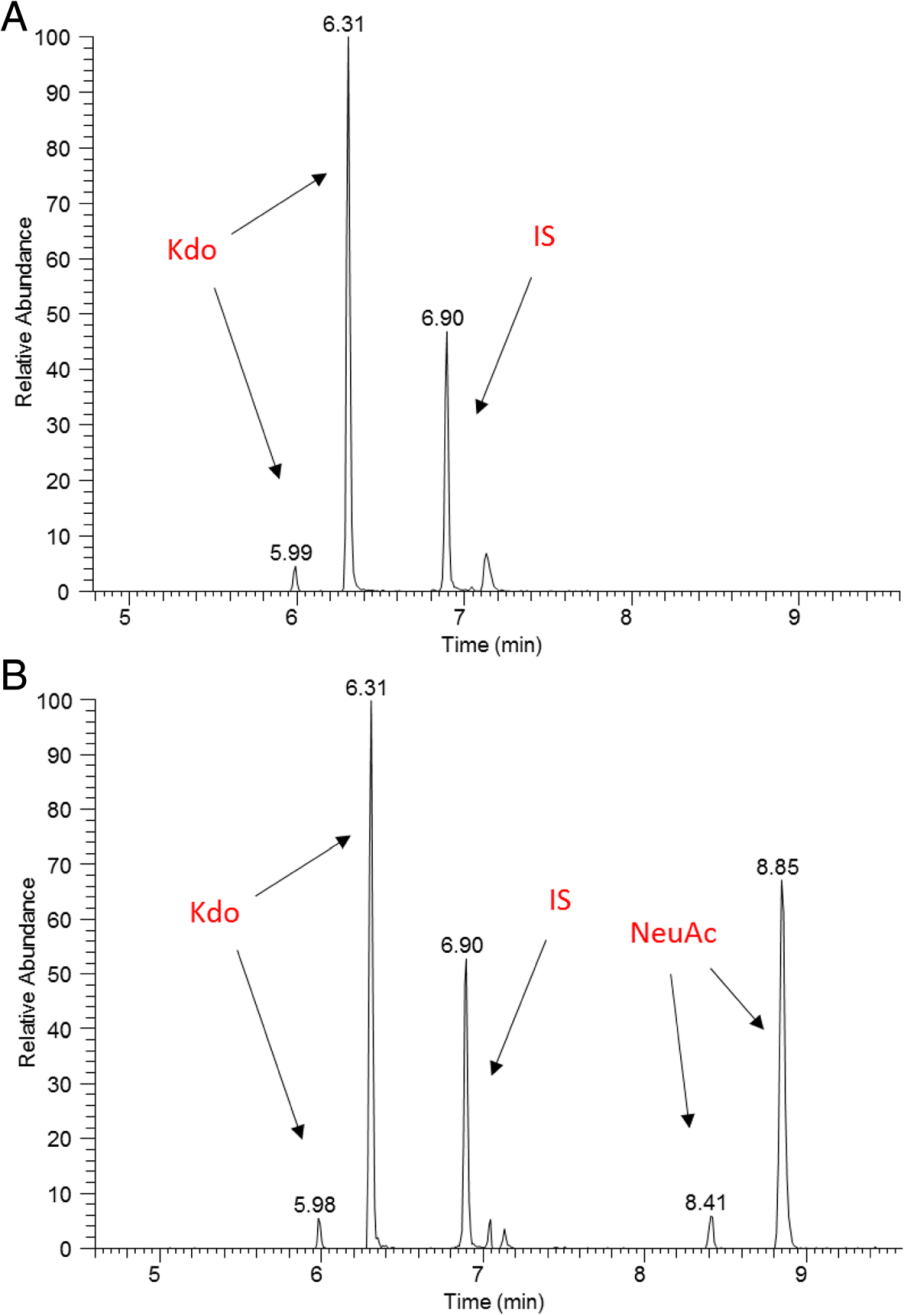
GLC-MS/MS analysis of NeuAc (sialic acid) content. **a** Tested *Salmonella* Hvittingfoss PCM 2946 strain; **b** Control: *Hafnia alvei* PCM 2386. MS/MS simultaneous analysis of Kdo (marker ion of m/z = 195), NeuAc (ion of m/z = 386), and perseitol (ion of m/z = 128) as an internal standard (IS)

Gas-liquid chromatography/tandem mass spectrometry analysis (GLC-MS/MS) of NeuAc content was performed in the preparations of whole bacteria. In this study the presence of NeuAc in bacterial cells in minimal medium were analyzed. All tested *Salmonella* strains were NeuAc negative. Figure 3 shows exemplary chromatograms of two samples: the control sample of *H. alvei* PCM 2386 with confirmed presence of NeuAc in the LPS structure [35] and one of the tested strains: *Salmonella* PCM 2946.

## Discussion

*Salmonella* as a pathogen is usually connected with gastrointestinal infection, often of zoonotic origin – mainly from wild or breeding birds. Our previous studies confirmed that the outer membrane structures of *S.* Enteritidis strains exhibiting high serum resistance play a crucial role in that phenomenon [36]. We have also shown, that these strains produce very long O-specific chain in LPS structure along with specific outer membrane proteins, what enhanced their pathogenicity [36]. Another studies have shown that some *Salmonella* O48 strains, which are frequently isolated from reptiles, use special virulence strategy predisposing them to the development of severe extraintestinal infection [37]. In the present work we analyzed the level of pathogenicity and phylogenetic structure of *Salmonella* strains isolated from reptiles with *S.* Enteritidis strains isolated from humans.

*S.* Enteritidis is a common foodborne pathogen, disseminated worldwide. The incidence of *S.* Enteritidis infections was increasing very fast in the 1990s and is now the most frequently reported *Salmonella* serovar around the world [38]. However, the number of reptile-associated *Salmonella* infections, often with fatal outcome, has been increasing together with popularity of handling reptiles as domestic pets [39]. Research focused on RAS were conducted e.g. in USA [40–42], Korea [43], Mexico [8, 44] or Malaysia [45]. The issue of reptiles as a reservoir of pathogenic *Salmonella* strains has been examined also in Europe, e.g. in Germany [17], Italy [46, 47], UK [48] or Sweden [49] where for years the *Salmonella* controlling and preventing program included special restrictions towards *Salmonella* strains originating from reptiles [50].

There are not many reports about *Salmonella* strains isolated from reptiles in Poland and the epidemiology of RAS/REPAS strains is unexplored and unknown. The presented study is one of the initial works about this epidemiological situation, connected with prevalence of *Salmonella* strains among exotic reptiles present in Poland. The majority of the research related with *Salmonella* strains isolated from reptiles include reptiles as domestic pets or wild reptiles free-living in Poland [30, 31], but in our research *Salmonella* strains were isolated from exotic reptiles belonging to the collection of Zoo in Wrocław, Poland. Reptiles in zoos often come from a custom control that capture smuggled and illegally imported animals taken from their natural environment. *Salmonella* serovars and other bacterial strains isolated from gastrointestinal tract of reptiles dwelled in zoos, could have an endemic character, being not previously reported in European countries.

Our results confirmed that exotic reptiles are common, asymptomatic carriers of *Salmonella* strains. From 84 samples collected from reptiles dwelled in Wrocław Zoo we isolated 15 *Salmonella* strains. Among them 4 isolated strains belonged to *Salmonella enterica* subsp. *diarizonae* and 3 to *Salmonella enterica* subsp. *salamae*. These *Salmonella* serovars are not typical for European region and have been imported together with reptiles from their exotic, native countries.

In the investigation of Piasecki et al. [30] *Salmonella* were found in 122 of 374 samples (32.6%). This research was focused on the occurrence of *Salmonella* strains in the natural microflora of exotic reptiles residing in Poland (zoos or private keepers). Also Zając et al. [31] determined the prevalence of *Salmonella* and other bacteria among 16 dead free-living snakes found in central Poland. *Salmonella* strains were detected in 87.5% of the tested animals what included 33 bacterial isolates representing 11 *Salmonella* serovars. The most frequent isolated serovars were *Salmonella enterica* subsp. *diarizonae* (IIIb) (*n* = 9) and *Salmonella enterica* subsp. *enterica* (*n* = 2) [31].

Results obtained in current paper are consistent with other research reports [10, 18, 49]. Similarly as in study carried out in Zagreb Zoo between 2009 and 2011 [10] *Salmonella* serovars typical for exotic countries, identified in Poland for the first time, belonging to subspecies *diarizonae* and *salamae* [32–34]. Moreover Wikström et al. isolated *S. enterica* subsp. *diarizonae* strains and *S. enterica* subsp. *enterica* Fluntern [49], which are occasionally isolated from reptiles but not detected in our study. In Antwerp Zoo in Belgium reptiles were carriers of *S. salamae*, *S. diarizonae*, *S. arizonae* and *S. houtenae* [51]. The team of Schröter revealed that about 81% of the tested snakes harbored various serovars of *S. enterica* subsp. *diarizonae* [18]. All these results confirm, that reptiles serve nowadays as vectors spreading exotic *Salmonella* serovars in new ecological niches including Europe.

Moreover, the comparative analysis of two *Salmonella diarizonae* strains isolated from invasive infections of humans, revealed that this *Salmonella* serotype pose a huge health-risk, as Giner-Lamia et al. [52] identified in genomes of *S. diarizonae* strains a number of genes responsible for high virulence.

In the present study we isolated from reptiles *Salmonella* serovars, which are well-known as human pathogens, such as *S.* Virchow, *S.* Amsterdam, *S.* Muenchen; that could be connected with the transmission of bacterial strains from humans to reptiles. Pedersen et al. [53] isolated *S*. Enteritidis and *S*. Typhimurium, which are commonly isolated from humans and often cause food contamination. In other studies *S.* Enteritidis, *S*. Typhimurium, *S*. Newport, *S*. Muenchen, *S*. Java and *S*. Pomona were reported as RAS/REPAS [39].

In the GLC-MS/MS analysis we did not detect the presence of NeuAc in the LPS structure among the tested *Salmonella* strains isolated from reptiles, what indicates, that none of these strains belonged to the O48 group.

Restriction analysis with PFGE, carried out during the presented work, showed a great diversity among *Salmonella* strains (*n* = 13) isolated from reptiles. Almost all tested strains had distinct restriction patterns, only two strains of *Salmonella* Amsterdam isolated from two different animals: Marginated tortoise (*Testudo marginata*) and Radiated tortoise (*Astrochelys radiate*) had completely the same PFGE profile. Probably it is a result of sharing the same bacterial flora between animals kept together, as transmission of *Salmonella* strains could occur via the faces in the water or food. Pees et al. [17] analyzed the phylogenetic structure of *Salmonella* strains isolated from reptiles kept in households in Germany with PFGE method. In that study PFGE profiles, obtained for all tested strains, were also diverse and the dominant PFGE type was not found as in our research [17].

Frequently one animal could carry more than one *Salmonella* serovar [54]. Zając et al. have found four different *Salmonella* serovars in a single snake [31]. Also in our study we have identified three various *Salmonella* serovars in one tested animal – Central bearded dragon (*Pogona vitticeps*). It is another reason why reptiles are efficient vectors for *Salmonella* strains: these animals are asymptomatic carriers even for more than one pathogenic bacterial strain, each of them can be easily transmitted to humans. Moreover, carrying several *Salmonella* serovars by one reptile could lead to an exchange of genetic material by horizontal gene transfer [55] what enhances the pathogenicity of *Salmonella* strains by an acquisition of new virulence genes or other genetic factors.

In the present project we compare *Salmonella* strains isolated from reptiles with *S.* Enteritidis isolated from humans with gastrointestinal disease. The results of PFGE analysis showed that tested *S.* Enteritidis strains are divided in three clusters. Minor differences in restriction patterns could be the result of genetic rearrangements that enables strains for adaptation to host organism and enhance their pathogenicity. However, the dominant PFGE types of *S.* Enteritidis in our study are similar to the dominant PFGE patterns obtained in analysis of clinical and environmental *S.* Enteritidis strains isolated in different parts of the world, e.g. Turkey, United States or Canada and Morocco [38, 56, 57].

Almost all known virulence genes (VGs), which are very significant in *Salmonella* pathogenicity were detected in all tested *S.* Enteritidis strains. Such a set of virulence genes enhance the successful host invasion, colonization and disease development, what could be a reason of worldwide dissemination of *S.* Enteritidis as the most frequently isolated clinical *Salmonella* serovar.

*Salmonella* strains isolated from reptiles show also high prevalence of tested VGs. Presented paper is the first study, where 17 virulence genes, typical for *Salmonella*, were detected among strains isolated from reptiles. Our studies indicate that *S*. Enteritidis strains have a high level of VGs presence, but none of the strains have the *cdtB* (host recognition/invasion) gene, which occurs in several *Salmonella* isolates from reptiles. On the other hand, none of the *Salmonella* strains isolated from reptiles have *spvB* (growth within host) and *pefA* (host recognition/invasion) genes, which occurs in all strains of *S*. Enteritidis. Skyberg et al. [58] determined the prevalence of these 17 VGs among *Salmonella* strains isolated from healthy birds [*n* = 80] and also from birds with infection [*n* = 76]. Results obtained by Skyberg’s group revealed, that all of tested bacterial strains from both groups of birds had following VGs: *spiA, pagC, msgA, invA, sipB, prgH, spaN, orgA, tolC, iroN, sitC*. Krawiec et al. [59] had used the same VGs set and determined its prevalence among *Salmonella* strains isolated from wild birds in Poland. In all isolated *S.* Typhimurium strains Krawiec at al. found following genes: *spiA*, *msgA*, *invA*, *lpfC* and *sifA*, additionally 94.45% of the tested bacterial isolates carried also the *sitC* and *sopB* virulence genes.

Our research showed that clinical strains of *S.* Enteritidis, which are the most common cause of salmonellosis throughout Europe, are not genetically homogeneous, we obtained three different genomic clusters for the tested 15 *S*. Enteritidis strains. The obtained results revealed the continuous adaptation of *S*. Enteritidis to the environment. In the presented study we proved that all tested *S*. Enteritidis strains have almost all tested virulence genes which are important from the point of infection.

The presence of a large number of virulence genes predisposes *S*. Enteritidis to be the most commonly isolated clinical strain in Europe. Nevertheless the tested *Salmonella* strains isolated from reptiles also show a wide diversity of the detected virulence genes.

## Conclusions

Exotic reptiles in Europe, can work as vectors introducing new strains of dangerous bacteria to the environment. Such bacteria could be recognized as a new, important epidemiological factor, very distinct from local endemic bacterial flora, especially in the face of widespread trade of reptiles around the world, and their presence in our household as pets. Our findings also highlight, that the knowledge about microflora of reptiles and appropriate hygienic conditions should be recommended for handling of reptiles. In addition, prevention of human infections requires proper education about personal hygiene. In comparison to *S.* Enteritidis, *Salmonella* strains isolated from reptiles are definitely more heterogeneous in phylogenetic view. *S.* Enteritidis as common and prevalent pathogen has few genetic patterns and is quite homogeneous.

## Methods

### Bacterial strains

The study was carried out on 15 clinical *S.* Enteritidis strains isolated from the feces of humans with symptoms of diarrhea in years 2012–2013 in Dialab Laboratory, Wrocław, Poland and 15 *Salmonella* strains isolated from feces of healthy reptiles from the Zoological Garden in Wrocław, Lower Silesia in Poland in years 2011–2014. All *Salmonella* strains used in this study were deposited in the Polish Collection of Microorganisms (PCM).

### Growth conditions

For genetic assays, the bacteria were cultured overnight at 37°C in Lysogeny Broth (LB) or nutrient agar (Biocorp). For gas-liquid chromatography mass spectrometry analysis (GLC-MS), the bacteria were cultivated in minimal medium [K_2_HPO_4_, KH_2_PO_4_, MgSO_4_, (NH_4_)_2_SO_4_, glucose, and NaCl (POCh, Poland)] needed in GLC-MS analysis.

### Identification of presumptive *Salmonella* strains with conventional methods

*Salmonella* strains were identified, using biochemical tests and serotyping with specific O and H antisera, and classified according to the Kauffmann-White-Le Minor scheme [2]. Serotyping of *Salmonella* strains isolated from reptiles was performed in the National Veterinary Research Institute (Puławy, Poland) and National *Salmonella* Centre (Gdańsk, Poland). A complete list of the tested strains is presented in Table 1.

### Identification with mass spectrometry methods

#### Sample preparation for MALDI-TOF MS analysis

Bacterial sample were prepared according to the manufacturer’s protocol (BrukerDaltonics, USA). Shortly: two to five bacterial colonies were suspended in water and precipitated with ethanol. After drying, equal volumes of 70% formic acid and acetonitrile were added and, after centrifugation, 1 μl of supernatant was transferred to ground steel MALDI plate for analysis, with α-cyano-4-hydroxy-cynnamic acid in 50% ethanol with 2,5% TFA used as a matrix. Identification of bacterial strains using MALDI–TOF MS Biotyper was conducted with the application of ultraflExtreme (BrukerDaltonics, USA). Spectra were recorded in the positive linear mode for a mass range of 2000–20.000 Da. Each spectrum was obtained by averaging 600 laser shots acquired from the automatic mode under control of FlexControl software ver. 3.4 (BrukerDaltonics, USA). The spectra were externally calibrated using an *E. coli* DH5-alpha standard (BrukerDaltonics, USA). Biotyper ver. 3.1 (MSP 4613) database software (BrukerDaltonics, USA) was used for the identification of bacterial isolates.

### Molecular identification of *Salmonella* isolates

#### DNA extraction

Bacterial DNA was extracted using commercially available Genomic Mini Kit (A&A Biotechnology, Poland) according to the manufacturer’s protocol from overnight (18–24 h, 37°C) culture.

#### 16S rRNA identification

To amplify the entire ~ 1500-bp region of the 16S rRNA gene universal primers were used: 16S_Start (5’AGAGTTTGATCMTGGCTCAG3’) and 16S_Stop (5’AAGGAGGTGWTCCARCC3’). In brief, samples underwent an initial denaturation of 5 min at 98°C and 35 cycles of 10 s at 98°C (denaturation), 30 s at 60°C (annealing), 45 s at 72°C (extension), followed by 5 min at 72°C (final extension). The products were sequenced (Sanger’s method). Sequences were aligned using DNA Baser v4.36.0. The isolate was identified using the EzTaxon server [60] on the basis of 16S rRNA sequence data.

#### Virulence genotyping

Strains were subjected to the testing of 17 virulence genes related to pathogenicity of *Salmonella*. The genes *invA, sipB, prgH, spaN, orgA, tolC, iroN, sitC, lpfC, sifA, sopB, pefA, spvB, spiA, pagC, cdtB* and *msgA* were targeted by three multiplex-PCR reactions using the protocol given below according to literature [58] with author’s modifications. The list of the primers used in this study (Genomed, Poland) and VGs functions are presented in Table 2. The cycling conditions for all three reactions were the same and set as follows: 95°C for 5 min and 30 cycles of denaturation (30 s, 94°C), annealing (30 s, 66.5°C), extension steps (2 min, 72°C), and final extension (10 min, 72°C). PCR amplifications of each type of reaction were performed with a DNA Thermal Cycler T100 (Bio-Rad, USA).

**Table 2:** Primers used in virotyping PCR reactions, with their sequence, size of ampliconsand biological function of targeted genes

Gene target	Primer sequence	Amplicon size (bp)	Function of gene
*invA*	F-CTGGCGGTGGGTTTTGTTGTCTTCTCTATT	1070	Host recognition/invasion
	R-AGTTTCTCCCCCTCTTCATGCGTTACCC		
*orgA*	F-TTTTTGGCAATGCATCAGGGAACA	255	Host recognition/invasion
	R-GGCGAAAGCGGGGACGGTATT		
*prgH*	F-GCCCGAGCAGCCTGAGAAGTTAGAAA	756	Host recognition/invasion
	R-TGAAATGAGCGCCCCTTGAGCCAGTC		
*spaN*	F-AAAAGCCGTGGAATCCGTTAGTGAAGT	504	Entry into nonphagocytic cells, killing of macrophages
	R-CAGCGCTGGGGATTACCGTTTTG		
*tolC*	F-TACCCAGGCGCAAAAAGAGGCTATC	161	Host recognition/invasion
	R-CCGCGTTATCCAGGTTGTTGC		
*sipB*	F-GGACGCCGCCCGGGAAAAACTCTC	875	Entry into nonphagocytic cells, killing of macrophages
	R-ACACTCCCGTCGCCGCCTTCACAA		
*sitC*	F-CAGTATATGCTCAACGCGATGTGGGTCTCC	768	Iron acquisition
	R-CGGGGCGAAAATAAAGGCTGTGATGAAC		
*pagC*	F-CGCCTTTTCCGTGGGGTATGC	454	Survival within macrophage
	R-GAAGCCGTTTATTTTTGTAGAGGAGATGTT		
*msgA*	F-GCCAGGCGCACGCGAAATCATCC	189	Survival within macrophage
	R-GCGACCAGCCACATATCAGCCTCTTCAAAC		
*spiA*	F-CCAGGGGTCGTTAGTGTATTGCGTGAGATG	550	Survival within macrophage
	R-CGCGTAACAAAGAACCCGTAGTGATGGATT		
*iroN*	F-ACTGGCACGGCTCGCTGTCGCTCTAT	1205	Iron acquisition
	R-CGCTTTACCGCCGTTCTGCCACTGC		
*sopB*	F-GGACCGGCCAGCAACAAAACAAGAAGAAG	220	Host recognition/invasion
	R-TAGTGATGCCCGTTATGCGTGAGTGTATT		
*lpfC*	F-GCCCCGCCTGAAGCCTGTGTTGC	641	Host recognition/invasion
	R-AGGTCGCCGCTGTTTGAGGTTGGATA		
*cdtB*	F-ACAACTGTCGCATCTCGCCCCGTCATT	268	Host recognition/invasion
	R-CAATTTGCGTGGGTTCTGTAGGTGCGAGT		
*sifA*	F-TTTGCCGAACGCGCCCCCACACG	449	Filamentous structure formation
	R-GTTGCCTTTTCTTGCGCTTTCCACCCATCT		
*pefA*	F-GCGCCGCTCAGCCGAACCAG	157	Host recognition/invasion
	R-GCAGCAGAAGCCCAGGAAACAGTG		
*spvB*	F-CTATCAGCCCCGCACGGAGAGCAGTTTTTA	717	Growth within host
	R-GGAGGAGGCGGTGGCGGTGGCATCATA		

#### Gel electrophoresis, visualization, and analysis of PCR amplification products

The amplified products from all types of the PCR reactions were resolved on a 2% or 0.8% agarose gel (Sigma-Aldrich, USA) and visualized with Midori Green DNA (Nippon Genetics, Germany) under UV light using a Gel Doc camera system (Bio-Rad, USA) and analyzed with Quantity One software (Bio-Rad, USA). PCR assays were repeated twice to confirm the correctness of the assignment of the investigated strains to their respective patterns.

#### Pulsed-field gel electrophoresis (PFGE)

All bacterial isolates were fingerprinted by the PFGE method, using the PulseNet protocol developed by Centers for Diseases Control and Prevention [61]. Chromosomal DNA was subjected to restriction analysis with application of XbaI enzyme (Thermo Fisher Scientific, USA). PFGE analysis was conducted with CHEF DR III PFGE apparatus (Bio-Rad, USA). DNA separation was performed with the following parameters: 1% agarose gel (Prona Agarose) on 0.5 M Tris–Borate–EDTA buffer at 14°C for 19 h at 6.0 V/cm (200 V). Pulse time was ranging of 2.2–63.8 s. The gels were stained with SYBR® Safe - DNA Gel Stain (Thermo Fisher Scientific, Germany) and band patterns were visualized under UV light and photographed using a Gel Doc camera system (Bio-Rad, USA). Molecular Weight Marker ProMega-Markers® Lambda Ladders was used for analysis (Promega, USA). PFGE patterns were analyzed via visual assessment and the dendrograms were generated with UPGMA method using on-line software http://insilico.ehu.es/.

### GLC-MS/MS analysis

#### Preparation of samples for GLC-MS/MS analysis

Samples for the analysis of NeuAc content were prepared according to Pawlak et al. [37]. In brief, bacteria and internal standard (perseitol, Koch-Light Laboratories Ltd., UK) were placed in a reaction tube and lyophilized. After lyophilization samples were methanolysed, evaporated and acetylated. After acetylation the samples were dried and dissolved in ethyl acetate (POCh, Poland). For GLC-MS/MS analysis 1 μl was taken. Samples of *Hafnia alvei* PCM 2386 with confirmed presence of NeuAc in the O-antigen [35] were used as a control.

### GLC-MS/MS analysis

Thermo FOCUS GC with ITQ 700 ion trap detector with external ionization (column: Restek, USA, Rxi – 5 ms, 30 m, 0.25 mm ID) was used for sample analysis by GLC-MS/MS. For MS/MS analysis primary ion *m/z* 446 was isolated and fragmented. The secondary fragment of *m/z* 386 was used for the quantitation of NeuAc derivative in the sample [28, 62].

## Additional file

Additional file 1: **Figure S1-S6.** The electrophoregrams of amplification products of the tested virulence genes. (PDF 332 kb)

## Data Availability

The datasets analyzed in the present study are available from the first and corresponding author on reasonable request. Additional supporting files can be found in the supplementary material section.

## Abbreviations

GLC-MS/MS: Gas-liquid chromatography/tandem mass spectrometry
LPS: Lipopolysaccharide
MALDI-TOF MS: Matrix assisted laser desorption/ionization time-of-flight mass spectrometry
NeuAc: Sialic acid
NTS: Nontyphoidal *Salmonella* strains
PCM: Polish Collection of Microorganisms
PCR: Polymerase chain reaction
PFGE: Pulsed-field Gel Electrophoresis
RAS: Reptile-associated salmonellosis
REPAS: Reptile-exotic-pet associated salmonellosis
SPI: *Salmonella* pathogenicity islands
VGs: Virulence-associated genes
