# Supplementary material for: Comparison of the phylogenetic analysis of PFGE profiles and the characteristic of virulence genes in clinical and reptile associated Salmonella strains
Source: BMC Vet Res. 2019 Sep 2;15:312. doi: 10.1186/s12917-019-2019-1 (PMC6721270; doi:10.1186/s12917-019-2019-1)

Figure S1. The electrophoregram of amplification products obtained for *S.* Enteritidis subjected to PCR reactions identifying following virulence genes: *spvB*, *spiA*, *pagC*, *cdtB* and *msgA.*

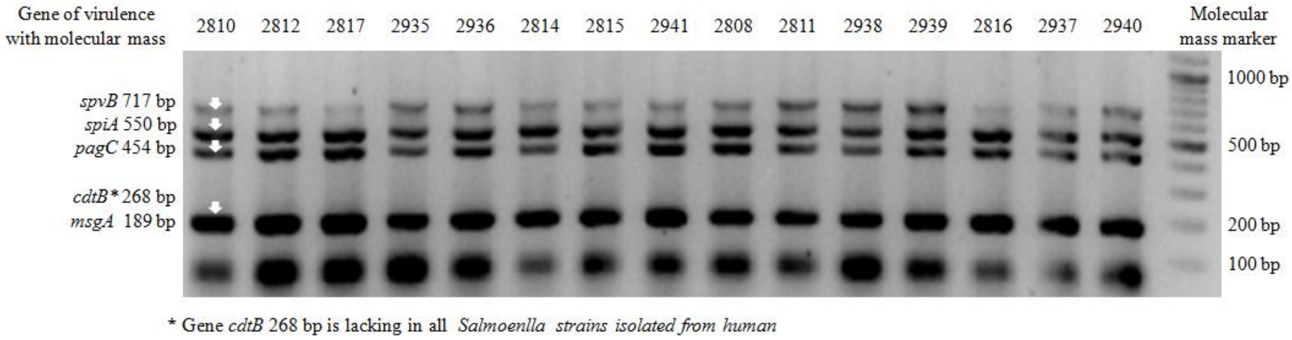

Figure S2. The electrophoregram of amplification products obtained for *S.* Enteritidis subjected to PCR reactions identifying following virulence genes: *iroN*, *sitC*, *lpfC*, *sifA*, *sopB* and *pefA.*

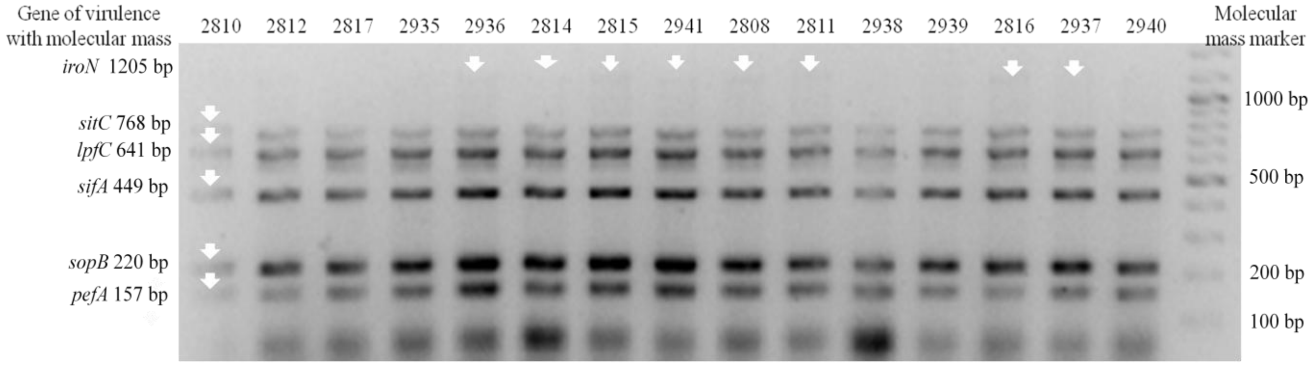

Figure S2. The electrophoregram of amplification products obtained for *S.* Enteritidis subjected to PCR reactions identifying following virulence genes: *invA*, *sipB*, *prgH*, *spaN*, *orgA* and *tolC.*

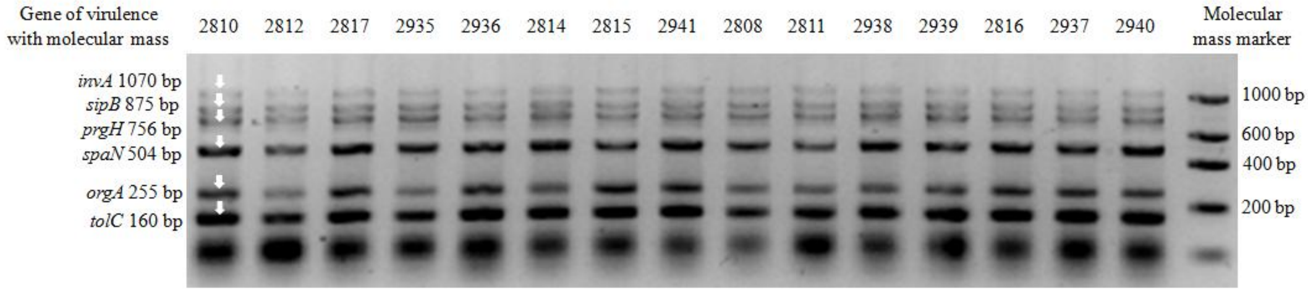

Figure S4. The electrophoregram of amplification products obtained for *Salmonella* isolated from reptiles subjected to PCR reactions identifying following virulence genes: *spvB*, *spiA*, *pagC*, *cdtB* and *msgA*.

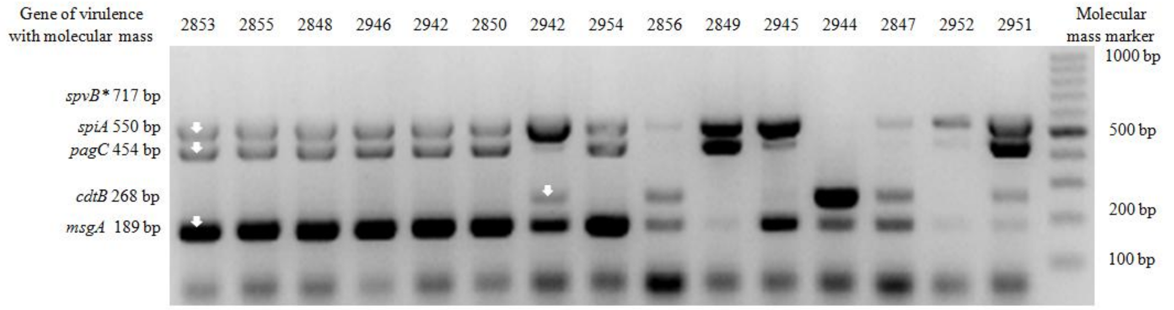

* Gene *spvB* 717 bp is lacking in all *Salmoenlla strains isolated from reptiles*

Figure S5. The electrophoregram of amplification products obtained for *Salmonella* isolated from reptiles subjected to PCR reactions identifying following virulence genes: *iroN*, *sitC*, *lpfC*, *sifA*, *sopB* and *pefA*.

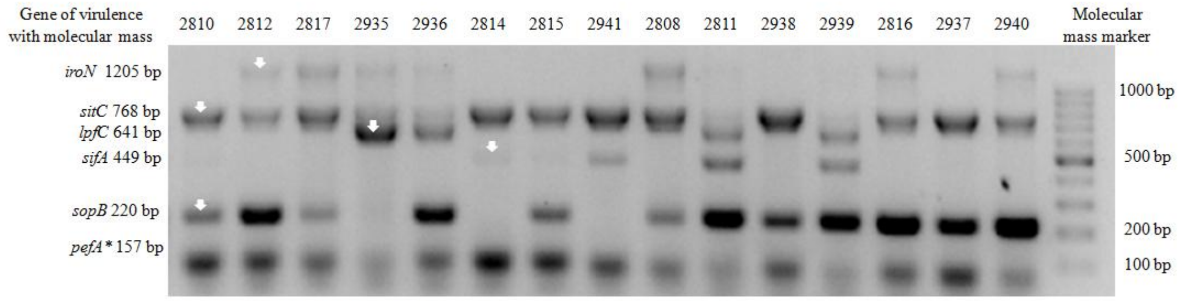

* Gene *pefA 157* bp is lacking in all *Salmoenlla strains isolated from reptiles*

Figure S6. The electrophoregram of amplification products obtained for *Salmonella* isolated from reptiles subjected to PCR reactions identifying following virulence genes: *invA*, *sipB*, *prgH*, *spaN*, *orgA* and *tolC*.

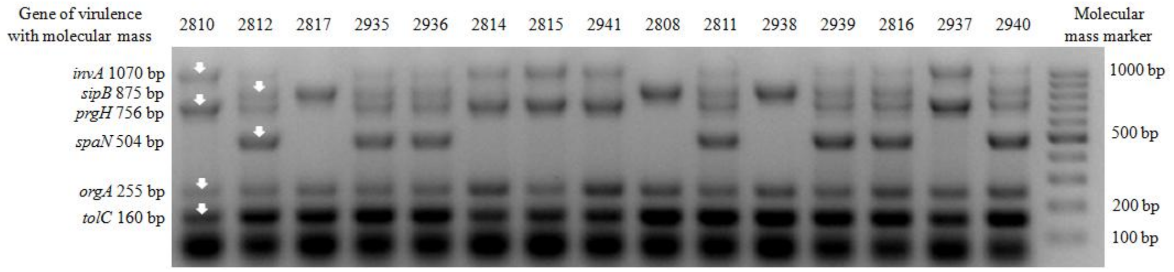

Supplement: Supplementary file 1 — Figure S1-S6. The electrophoregrams of amplification products of the tested virulence genes. (PDF 332 kb) [file 12917_2019_2019_MOESM1_ESM.pdf]
